# Supplementary material for: Interaction of genetic variants activates latent metabolic pathways in yeast
Source: Nat Commun. 2025 Aug 27;16:8014. doi: 10.1038/s41467-025-63306-4 (PMC12391433; doi:10.1038/s41467-025-63306-4)
Supplement: Supplementary file 1 — Supplementary Information [file 41467_2025_63306_MOESM1_ESM.pdf]

SUPPLEMENTARY INFORMATION

Interaction of Genetic Variants Activates Latent Metabolic Pathways in Yeast

Srijith Sasikumar[1,2,3], Shannara Taylor Parkins[4], Suresh Sudarsan[4], Himanshu Sinha[1,2,3,*]

[1] Systems Genetics Lab, Department of Biotechnology, Bhupat and Jyoti Mehta School of Biosciences, Indian Institute of Technology Madras, Chennai, India

[2] Centre for Integrative Biology and Systems Medicine (IBSE), Indian Institute of Technology Madras, Chennai, India

[3] Wadhwani School of Data Science and Artificial Intelligence (WSAI), Indian Institute of Technology Madras, Chennai, India

[4] The Novo Nordisk Foundation Centre for Biosustainability, Technical University of Denmark, Lyngby, Denmark

[*] Corresponding author: Himanshu Sinha sinha@iitm.ac.in

This supplementary information file includes

Supplementary Methods

Supplementary Note 1

Supplementary Tables 1 and 2

Supplementary Figures

SUPPLEMENTARY METHODS

RNA Extraction Protocol

RNA was extracted from yeast cells using Qiagen RNeasy mini kit (Cat No.74106). The cell pellet was resuspended in buffer RLT and incubated at room temperature for 5 min to achieve complete lysis. The lysate mixed with half volume of absolute alcohol was loaded into the RNeasy spin column placed in a 2 ml collection tube. The tubes were centrifuged at 8,000 rpm for 1 min, and the flow-through was discarded. On column DNase I (Cat No.79254), treatment and subsequent column washes were performed according to the manufacturer's protocol. RNA was eluted from the column using nuclease-free water.

The concentration and purity of RNA were quantified using a Nanodrop Spectrophotometer (Thermo Scientific; 2000). The integrity of RNA in the samples was assessed on Tapestation (Agilent). RNA concentration was quantified using the Qubit RNA HS assay kit (Q32855).

Library Preparation and Sequencing

RNA sequencing libraries were prepared with Illumina-compatible NEBNext® Ultra™ II Directional RNA Library Prep Kit (New England BioLabs, MA, USA) at Genotypic Technology Pvt. Ltd., Bangalore, India.

100-500 ng of total RNA was taken for mRNA isolation, fragmentation, and priming. Fragmented and primed mRNA was further subjected to first-strand synthesis followed by second-strand synthesis. The double-stranded cDNA was purified using NEBNext sample purification beads. Purified cDNA was end-repaired, adenylated, and ligated to Illumina adapters as per NEBNext® Ultra™ II Directional RNA Library Prep protocol, followed by second-strand excision using the USER enzyme at 37 ˚C for 15 minutes.

Illumina Universal Adapters used in the study were:

`5′-AATGATACGGCGACCACCGAGATCTACACTCTTTCCCTACACGACGCTCTTCCGATCT-3′`
and

Index Adapter:

`5′-GATCGGAAGAGCACACGTCTGAACTCCAGTCAC [INDEX] ATCTCGTATGCCGTCTTCTGCTTG-3′.`

[INDEX] – Unique sequence to identify sample-specific sequencing data.

Adapter-ligated cDNA was purified using NEBNext beads and was subjected to 11 cycles for Indexing-(98˚C for 30 sec, cycling (98˚C for 10sec, 65˚C for 75sec) and 65˚C for

5min) and enriched the adapter-ligated fragments. Final PCR products (sequencing library) were purified with NEBNext beads, followed by a library quality control check. Illumina-compatible sequencing libraries were quantified by Qubit fluorometer (Thermo Fisher Scientific, MA, USA), and fragment size distribution was analyzed on Agilent 2200 TapeStation.

Proteomic sample processing

Cell pellets of *S. cerevisiae* were lysed in 6 M guanidinium·HCl, 5 mM tris(2-carboxyethyl) phosphine, 10 mM chloroacetamide, and 100 mM Tris·HCl (pH = 8.5), disrupted mechanically, and heated to 99°C. After centrifugation, the cell-free lysates were diluted with 50 mM ammonium bicarbonate and subjected to a bicinchoninic acid (BCA) assay to estimate protein concentrations. Trypsin and LysC digestion mix (Promega) was added to 20 µg protein of each sample and incubated for 8 hours. Trifluoroacetic acid was added to halt digestion, and the samples were desalted using C18 resin (Empore, 3M) before HPLC-MS analysis. HPLC-MS analysis of the samples was performed on an Orbitrap Exploris 480 instrument (Thermo Fisher Scientific), preceded by an EASY-nLC 1200 HPLC system (Thermo Fisher Scientific). For each sample, 0.5 µg of peptides were captured on a 2 cm C18 trap column (Thermo Fisher 164946). Subsequently, separation was executed using a 70 min gradient from 8% (v/v) to 48% (v/v) of acetonitrile in 0.1% (v/v) formic acid on a 15 cm C18 reverse-phase analytical column (Thermo EasySpray ES904) at a flow rate of 250 nl min^{-1}. For data-independent acquisition, the mass spectrometer was run with the HRMS1 method as previously described[1], preceded by the FAIMS Pro Interface (Thermo Fisher Scientific) with a compensation voltage (CV) of -45 V, and any modifications are mentioned below. Full MS1 spectra were collected at a resolution of 120,000 and a scan range of 400-1,000 m/z, with the maximum injection time set to auto. MS2 spectra were obtained at a resolution of 60,000, with the maximum injection time set to auto and the collision energy set to 32. Each cycle consisted of three DIA experiments, each covering a range of 200 m/z with a window size of 6 m/z and a 1 m/z overlap, while a full MS scan was obtained between experiments.

Metabolomic sample processing and analysis:

For LC-MS/MS analysis, dried extracts were reconstituted in 200 µl of LC-MS-grade water containing 0.1% formic acid. Samples were vortexed for 30 seconds, spun briefly, and sonicated for 2 min. After centrifugation at 14,000 × g for 10 minutes at 4°C, the supernatant was transferred to a fresh tube and diluted 5-fold with 0.1% formic acid. A 10 µL aliquot was injected into the LC-MS system.

Chromatographic separation was performed using an Agilent 1290 Infinity II UHPLC system equipped with a C18 column (4 µm, 4.6 × 150 mm). The mobile phases consisted of water with 0.1% formic acid (A) and methanol with 0.1% formic acid (B) at a 0.5 ml/min

flow rate. The column oven and autosampler were maintained at 45 °C and 4 °C, respectively. The following gradient was applied: 0–3 min, 2% B; 3–12 min, linear increase to 35% B; 12–15 min, linear increase to 90% B; 15–16 min, 90% B; 16–16.1 min, decrease to 5% B; 16.1–20 min, re-equilibration at 5% B.

Detection was performed on an Agilent 6495 Triple Quadrupole mass spectrometer operating in positive ionization mode using multiple reaction monitoring (MRM). Data acquisition and quantification were conducted using Agilent MassHunter Workstation Quantitative Analysis software (version 10.1). To monitor instrument performance and ensure data quality, three pooled quality control (QC) samples were prepared by combining equal aliquots from all experimental samples and were analyzed at the beginning, middle, and end of the run. The coefficient of variation (CV) for each amino acid was calculated and ensured that the CV remained below 15% for all analytes (Supplementary Data 9).

Integrating protein expression data into a yeast genome-scale metabolic model

The protein constraint metabolic models for each strain and their respective time phases were generated by integrating the protein expression data into the latest genome-scale metabolic model of yeast (Yeast9)[2] (https://github.com/SysBioChalmers/yeast-GEM) using the model extraction method iMAT[3] using lower threshold as the minimum value of the expression array and upper threshold as the minimum value of top 10 % of reaction's expression array for that particular strain and time point. The bounds of exchange reactions of the GEM were changed to reflect the early stages of the sporulation state appropriately, as described in our previous study[4]. For this, we restricted the intake of glucose and nitrogen (Lower bound = Upper bound = 0 [mmol/ (g DW h)]) while allowing an unrestricted supply of oxygen and acetate (Lower bound = -1000 [mmol/ (g DW h)]). The metabolic heterogeneity between the context-specific models was studied by calculating the Jaccard index between the models.

Genome-scale differential flux analysis to predict upregulated and downregulated reactions

The optGpSampler[5] in COBRApy version 0.10[6] was used to sample 10,000 flux solutions with a thinning factor set to 100 on all the generated SNP and time-specific metabolic models.

Let X_{SNP} and X_{SS} be the flux distributions of a particular reaction in SNP-specific (MM, TT, and MMTT) and SS models for each time point (0h and 2h30m). The flux change was calculated for each reaction as follows:

$$Flux\ change = \frac{\bar{X}_{SNP} - \bar{X}_{SS}}{|\bar{X}_{SNP} + \bar{X}_{SS}|}$$

where, $\bar{X}_{SNP}$ and $\bar{X}_{ss}$, the arithmetic means of the flux distributions for a given reaction in SNP and null model, respectively.

To identify significantly altered reactions in each SNP-specific model compared to the null model, we employed a two-sided Kolmogorov-Smirnov test with a significance threshold 0.05, adjusted for multiple comparisons using the Benjamini-Hochberg method. From the set of dysregulated reactions, we classified those with a flux change greater than 0.82 as upregulated and those with a flux change below -0.82 as downregulated[4,7], corresponding to a tenfold increase or decrease in flux in the SNP model relative to the null model. The outline of steps involved in generating context-specific models and GS-DFA analysis is shown in Supplementary Fig. 18A.

SUPPLEMENTARY NOTE 1

We constructed context-specific metabolic models for the SS, MM, TT, and MMTT yeast strains at two time points: 0 h and 2 h 30 min. This was achieved by integrating SNP-specific protein abundance data into the Yeast GEM (Yeast9) using iMAT (Integrative Metabolic Analysis Tool). iMAT categorizes protein expression levels into high, moderate, and low, creating a subnetwork enriched with reactions driven by highly expressed proteins while minimizing the inclusion of reactions with low expression. It aims to maximize the consistency between reaction activity and protein expression, assigning non-zero flux to active reactions and zero to inactive ones. The SNP-specific models generated by iMAT, including the number of genes, reactions, and metabolites in each, are summarized in Supplementary Fig. 18B.

We calculated the Jaccard similarity index to evaluate the variability among these models. We observed that the models at 0 h (SS-0h, MM-0h, TT-0h, and MMTT-0h) and the 2h30m model of the low-sporulating strain (SS-2h30m) are highly similar. In contrast, the models for high-sporulating strains at 2 h 30 min (MM-2h30m, TT-2h30m, and MMTT-2h30m) exhibited high similarity (Supplementary Fig. 18C).

We then focused on identifying reactions upregulated in the MM, TT, and MMTT strains compared to the SS strain at both time points (Supplementary Data 10). Specifically, we investigated the flux through the arginine biosynthesis pathway, as we had previously shown that the MMTT strain exhibits unique regulation of this pathway, with increasing allocation during sporulation. Additionally, we aimed to identify pathways uniquely enriched in the MMTT strain, potentially due to genetic interactions between the *MKT1*^{89G} and *TAO3*^{4477C} SNPs.

Our analysis revealed that intracellular flux through the arginine biosynthesis pathway, particularly Argininosuccinate lyase (*ARG4*), is inactive at 0 hours in all strains but becomes active in the MM-2h30, TT-2h30, and MMTT-2h30 models while remaining inactive in the SS-2h30m model. This suggests that *ARG4* flux may be critical for efficient sporulation (Supplementary Fig. 19A, Supplementary Fig. 19B). Furthermore, flux enrichment analysis of upregulated reactions identified using GS-DFA revealed that the steroid biosynthesis pathway is uniquely enriched in the MMTT strain (adjusted p-value = 7.59E-08). The intracellular flux distribution of key reactions in the steroid biosynthesis pathway is illustrated in Supplementary Fig. 20.

SUPPLEMENTARY TABLE

Supplementary Table 1: List of strains used in the study

Strain identifier	Strain	Parent strain	Reference
	S288c		Deutschbauer and Davis[8]
	SK1		Deutschbauer and Davis [8]
SHS306	MM (*MKT1*-89G (a/alpha))	SHS304 x SHS305 (M strain)	Gupta et al.[9]
SHS327	TT (*TAO3*-4477C (a/alpha))	SHS325 x SHS326 (clean BC3T strain)	Gupta et al.[10]
SHS884	MmTt strain [*MKT1*(89G)/*MKT1*(89A) *TAO3*(4477C)/*TAO3*(4477G)]	by mating M strain and T strain	This study
SHS885	MT strain (a)	by sporulating MmTt strain	This study
SHS886	MT strain (alpha)	by sporulating MmTt strain	This study
SHS887	MMTT strain (diploid)	by crossing SHS885 and SHS886	This study
SHS888	S288c, Δ*arg4*::HygMX4 (a)	S288c (a)	This study
SHS889	M strain, Δ*arg4*::HygMX4 (alpha)	M strain (alpha)	This study
SHS890	T strain, Δ*arg4*::HygMX4 (a)	T strain (a)	This study
SHS891	MT strain, Δ*arg4*::HygMX4 (a)	MT strain (a)	This study
SHS892	S288c, Δ*arg56*::HygMX4 (a)	S288c (a)	This study
SHS893	M strain, Δ*arg56*::HygMX4 (alpha)	M strain (alpha)	This study
SHS894	T strain, Δ*arg56*::HygMX4 (a)	T strain (a)	This study
SHS895	MT strain, Δ*arg56*::HygMX4 (a)	MT strain (a)	This study
SHS896	S288c, Δ*arg4*::HygMX4 (Diploid)	S288c (a)	This study
SHS897	MM strain, Δ*arg4*::HygMX4 (Diploid)	M strain (alpha)	This study
SHS898	TT strain, Δ*arg4*::HygMX4 (Diploid)	T strain (a)	This study

SHS899	MMTT strain, Δ*arg4*::HygMX4 (Diploid)	MT strain (a)	This study
SHS900	S288c, Δ*arg56*::HygMX4 ((Diploid)	S288c (a)	This study
SHS901	MM strain, Δ*arg56*::HygMX4 (Diploid)	M strain (alpha)	This study
SHS902	TT strain, Δ*arg56*::HygMX4 (Diploid)	T strain (a)	This study
SHS903	MMTT strain, Δ*arg56*::HygMX4 (Diploid)	MT strain (a)	This study

Supplementary Table 2: List of primers used in the study

Primer name	Primer sequence 5'-3'	Description
HS3501	GCTCAAAAGCAGGTAACTATATAACAAGAC TAAGGCAAACcagctgaagcttcgtacgct	FP *ARG4* deletion
HS3502	CCAGACCTGATGAAATTCTTGCGCATAACGTCGCCA TCTGcataggccactagtggatctg	RP *ARG4* deletion
HS3503	ACGTATTTTAGCCTGGTTGT	FP with BN72 (Junction amplification *ARG4*)
HS3504	ATCCTGCAGATTGGGTCACA	RP with JM37 (Junction amplification *ARG4*)
HS3505	AGAAAGAGAGATCTGAAGTGAGAAATAAGT CTGCATCATTcagctgaagcttcgtacgct	FP *ARG56* deletion
HS3506	GTCTCATGTGACTGAGCTGCAGATCTACTATACACA GTGAcataggccactagtggatctg	RP *ARG56* deletion
HS3507	GGAAAGGTATCTGGGAAAGG	FP with BN72 (Junction amplification *ARG56*)
HS3508	AAACGCGTAAACAAGCTCGT	RP with JM37 (Junction amplification *ARG56*)
HS1013	CTGAATAATTGTACCCTGGA	Fwd primer to check *MKT1* 89A SNP with HS1015
HS1014	CTGAATAATTGTACCCTGGG	Fwd primer to check *MKT1* 89G SNP with HS1015
HS1015	GTTGAAACCAAGAGGAGTAA	Common reverse primer with HS1013 and HS1014
HS1016	TGATCTACTTTCAGCTGTTG	Forward primer to check *TAO3* 4477G SNP with HS1018
HS1017	TGATCTACTTTCAGCTGTTC	Forward primer to check *TAO3* 4477C SNP with HS1018
HS1018	GCTAAAGGAACCATGTATTT	Common reverse primer with HS1016 and HS1017

HS1127	CTCCACTTCAAGTAAGAGTTTGGGT	forward primers specific to the MATa
HS1128	TTACTCACAGTTTGGCTCCGGTGT	forward primers specific to the MATalpha
HS1129	GAACCGCATGGGCAGTTTACCTTT	common reverse primer to MAT alleles

SUPPLEMENTARY FIGURES

A

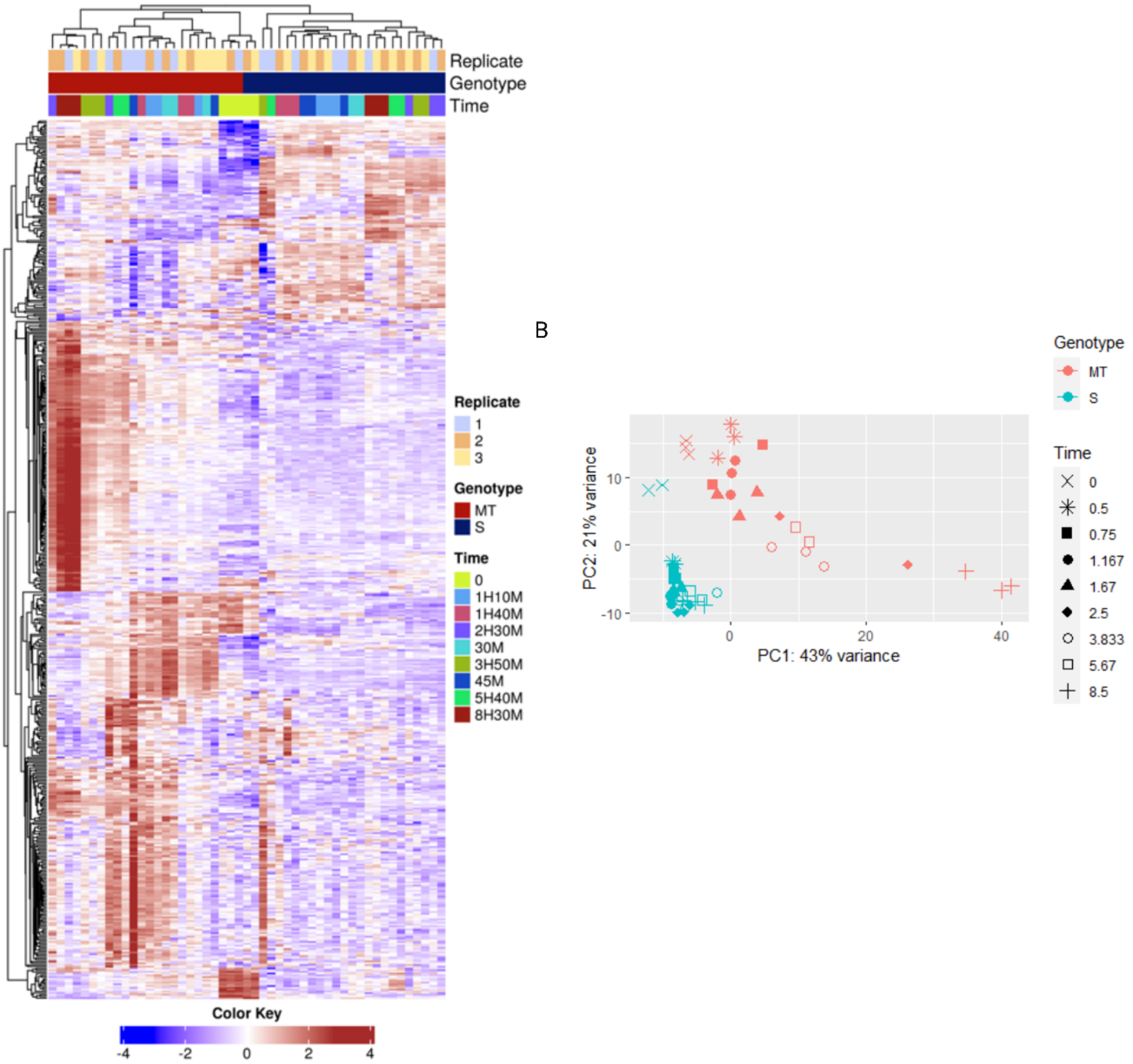

Supplementary Fig. 1: Distinct temporal transcriptional profiles in SS and MMTT strains during sporulation. (A) Heatmap representing the hierarchically clustered gene expression levels of SS and MMTT strains across 9 time points for the 300 most variable genes. (B) PCA plot based on the 300 most variable genes. The raw data for this heatmap is available on GEO under the accession number GSE278267 [https://www.ncbi.nlm.nih.gov/geo/query/acc.cgi?acc=GSE278267].

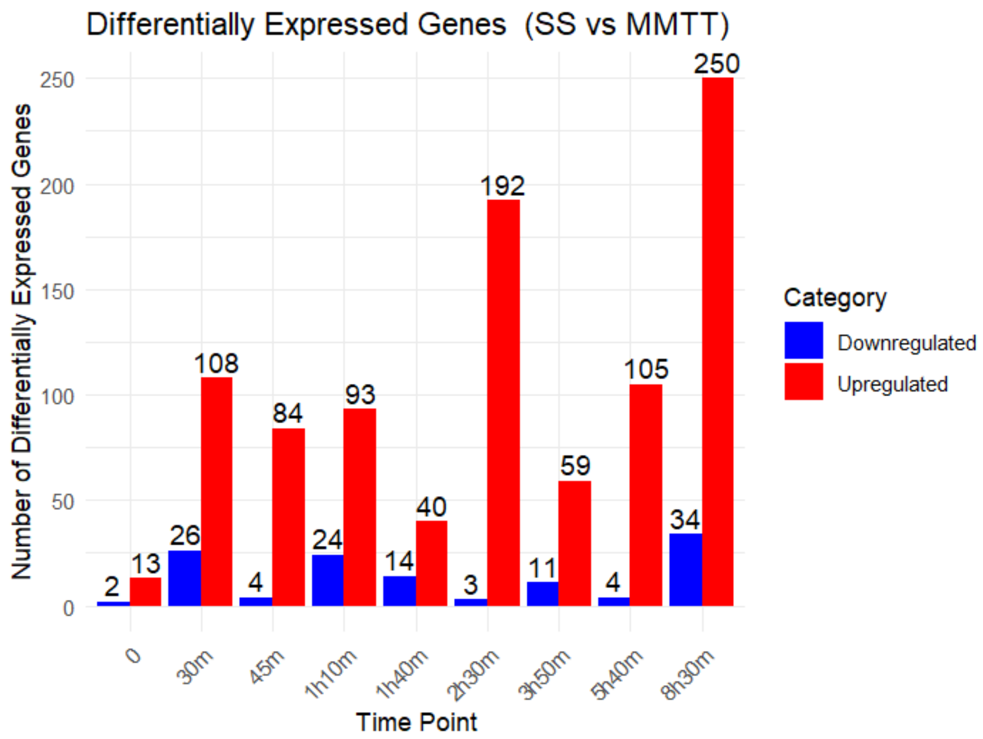

Supplementary Fig. 2: The number of differentially expressed genes in the MMTT strain compared to the SS strain at each time point during sporulation. DEGs were identified using DESeq2 with Wald test (two-sided), applying an adjusted p-value < 0.05 (Benjamini–Hochberg correction) and $|\log_2 \text{fold change}| > 2$. The source data is available in Supplementary Data 1.

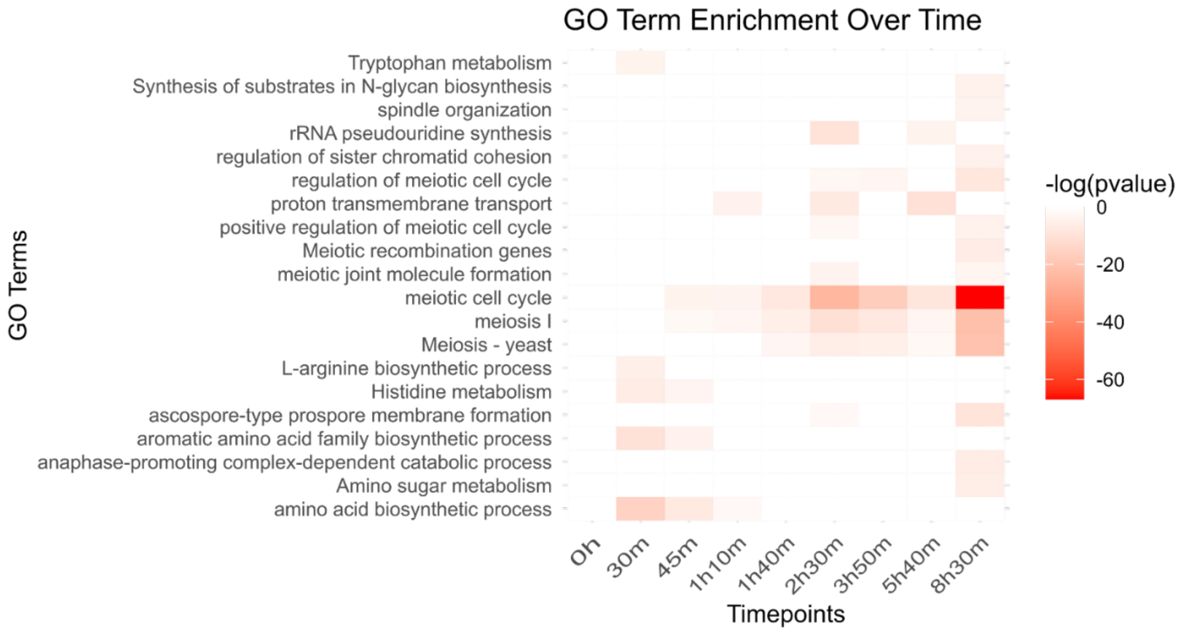

Supplementary Fig. 3: GO enrichment analysis for upregulated genes in the MMTT strain across each time point compared to the SS strain. The heatmap represents the p-value of each GO term. Enrichment p-values were calculated using a one-sided cumulative hypergeometric test and corrected for multiple comparisons using the Benjamini–Hochberg method. Source data are provided as a Source Data file.

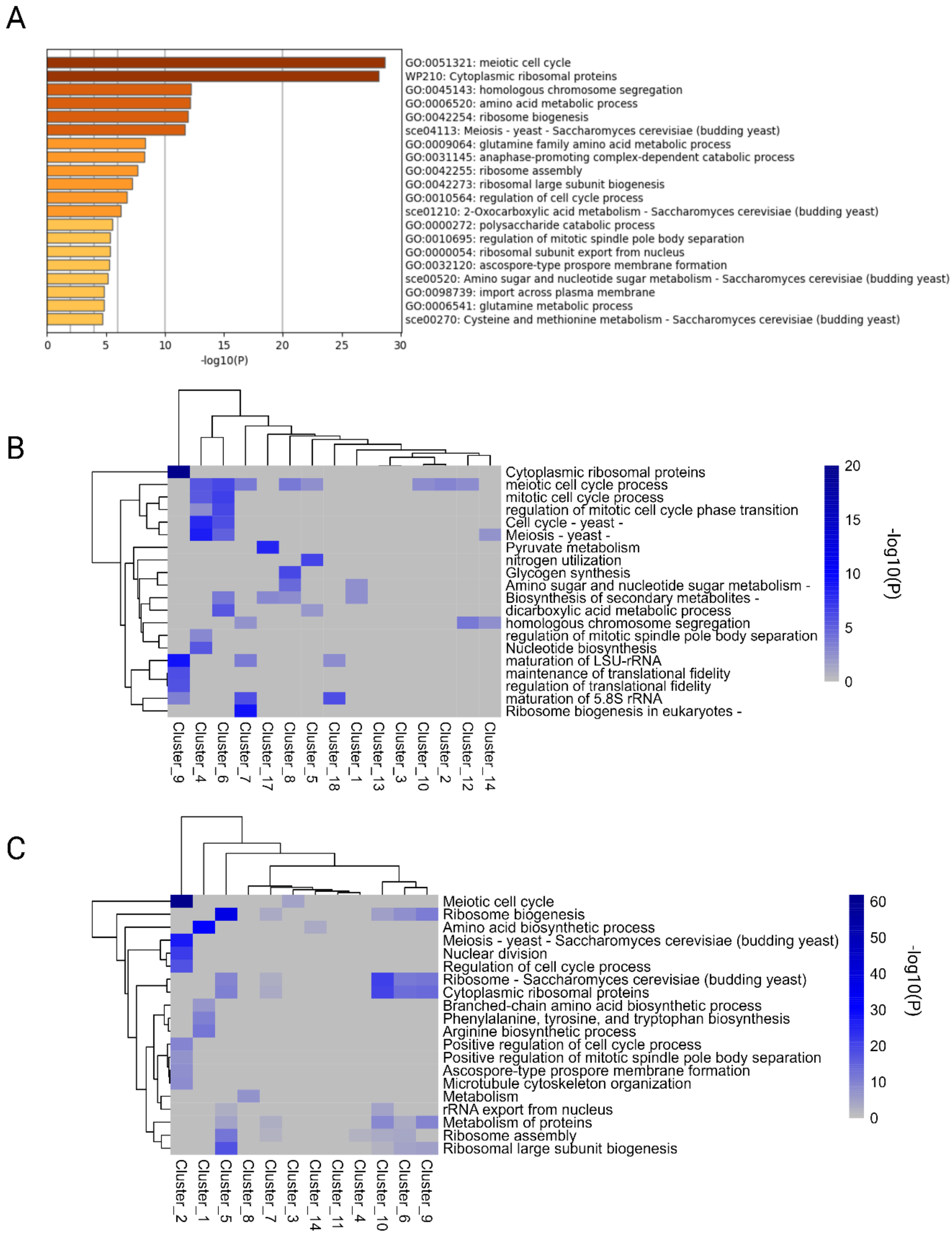

Supplementary Fig. 4: Metascape enrichment analysis reveals distinct pathway enrichment patterns between SS and MMTT strains over time. (A) Gene Ontology (GO) enrichment analysis was performed using Metascape on 1,080 differentially expressed genes (DEGs) identified between SS and MMTT strains across time points using the DESeq2 likelihood ratio test (LRT). DEGs were defined based on an adjusted p-value < 0.05 (Benjamini–Hochberg correction). (B–C) Heatmaps showing GO term enrichment

results for SS (B) and MMTT (C) strains. Rows represent GO terms and columns correspond to DPGP-derived gene expression clusters. Enrichment p-values were calculated using a one-sided cumulative hypergeometric test and corrected for multiple comparisons using the Benjamini–Hochberg method. Hierarchical clustering was applied to GO terms and gene clusters to reveal distinct temporal patterns. Source data are provided as a Source Data file.

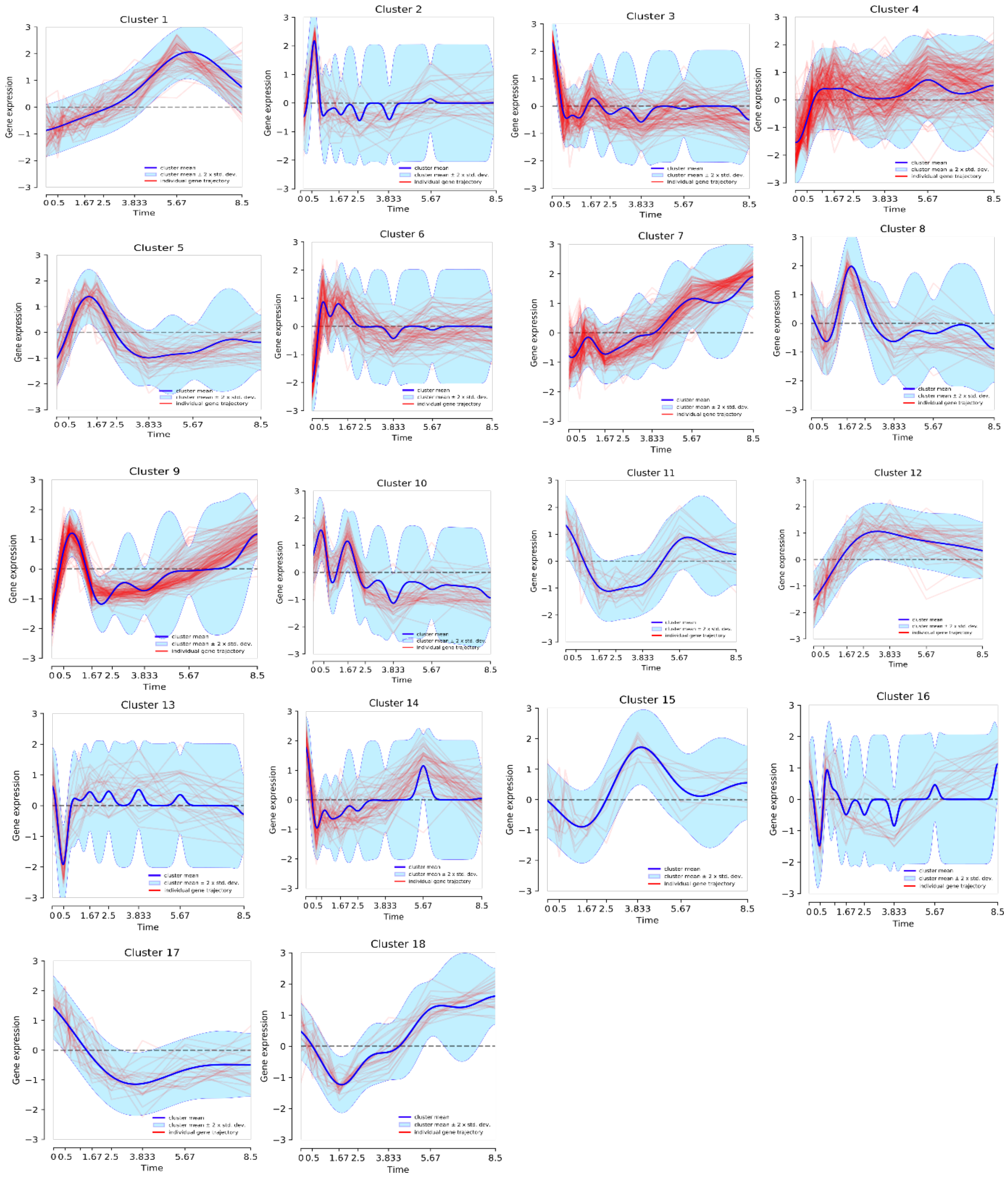

Supplementary Fig. 5: (A) Temporal gene expression trajectories of 18 clusters of SS train obtained through DPGP clustering on gene expression profiles of DEGs obtained from the DESeq2 LRT method. Source data are provided as a Source Data file.

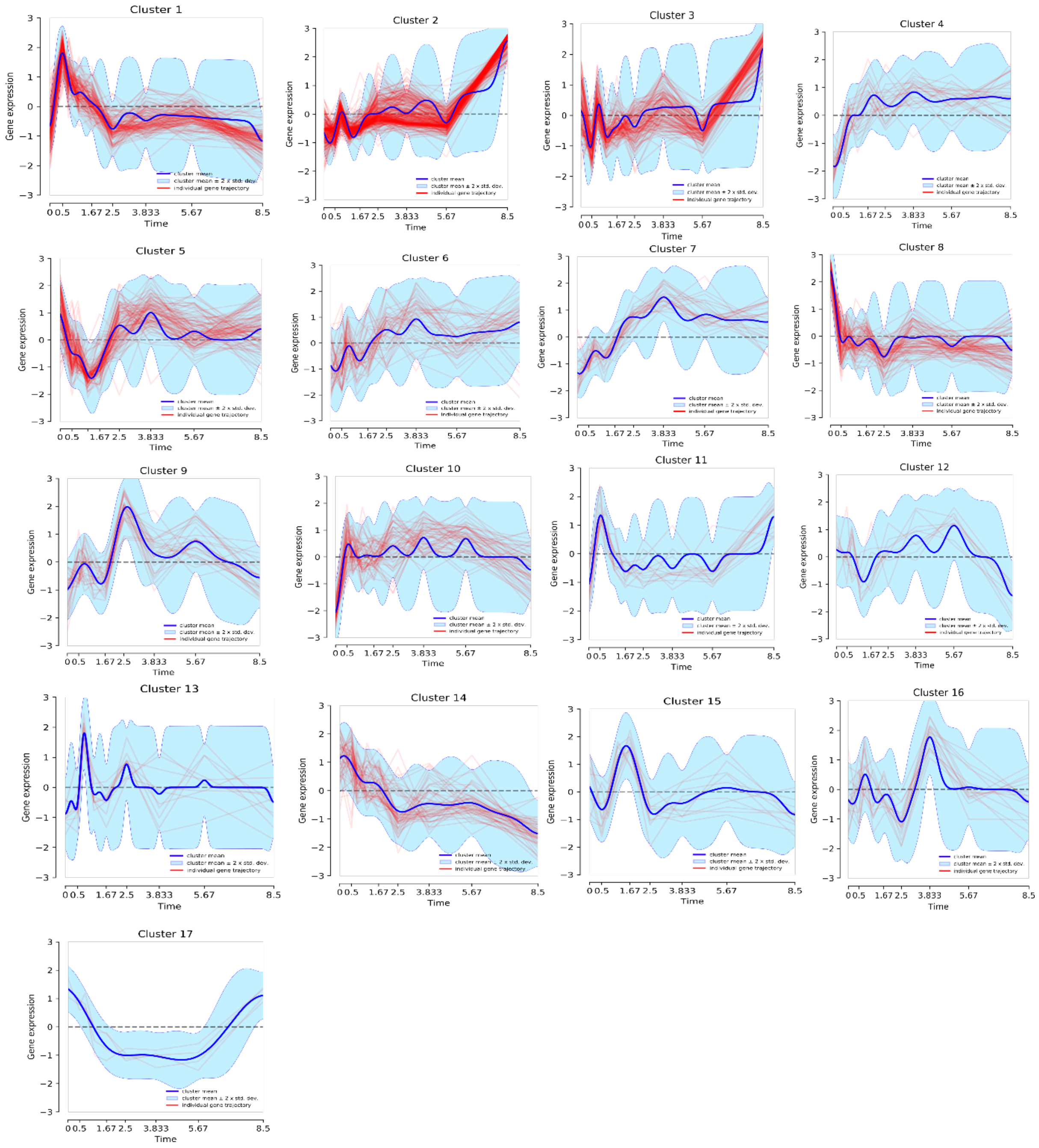

Supplementary Fig.5 (B) Temporal gene expression trajectories of 17 clusters of MMTT train obtained through DPGP clustering on gene expression profiles of DEGs obtained from the DESeq2 LRT method. Source data are provided as a Source Data file.

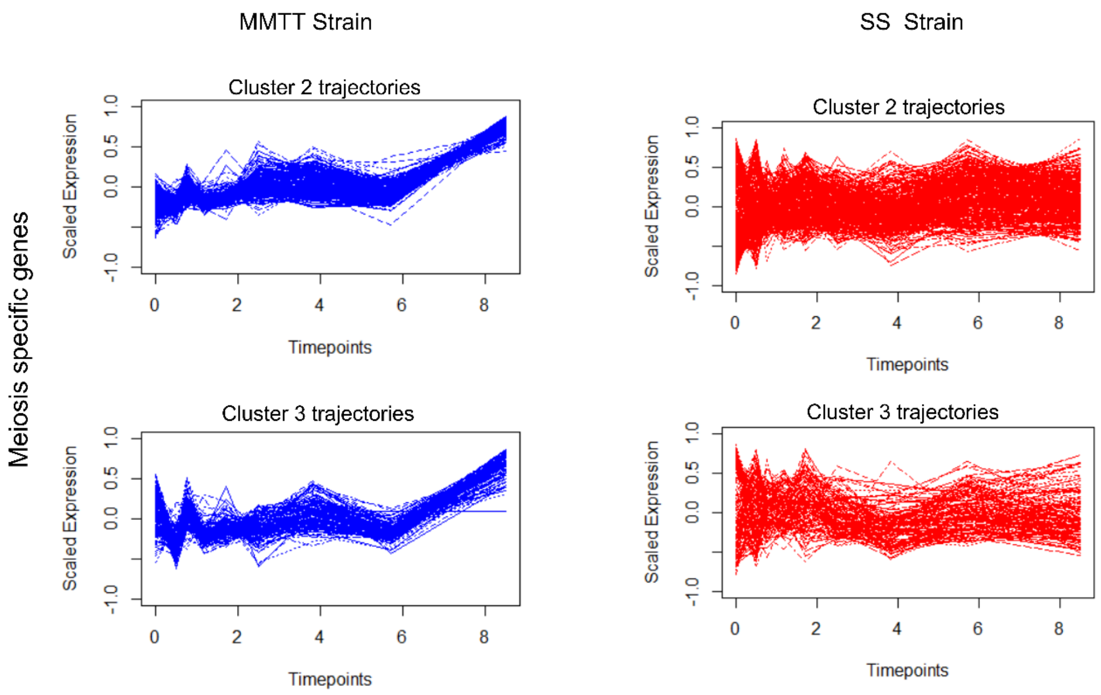

Supplementary Fig. 6: Temporal gene expression trajectories of genes in Clusters 2 and 3 of the MMTT train enriched for meiosis-specific pathways and their trajectories in the SS strain. The genes in each cluster are given in Supplementary Data 3. The source data is the same as in Supplementary Fig. 5A-B.

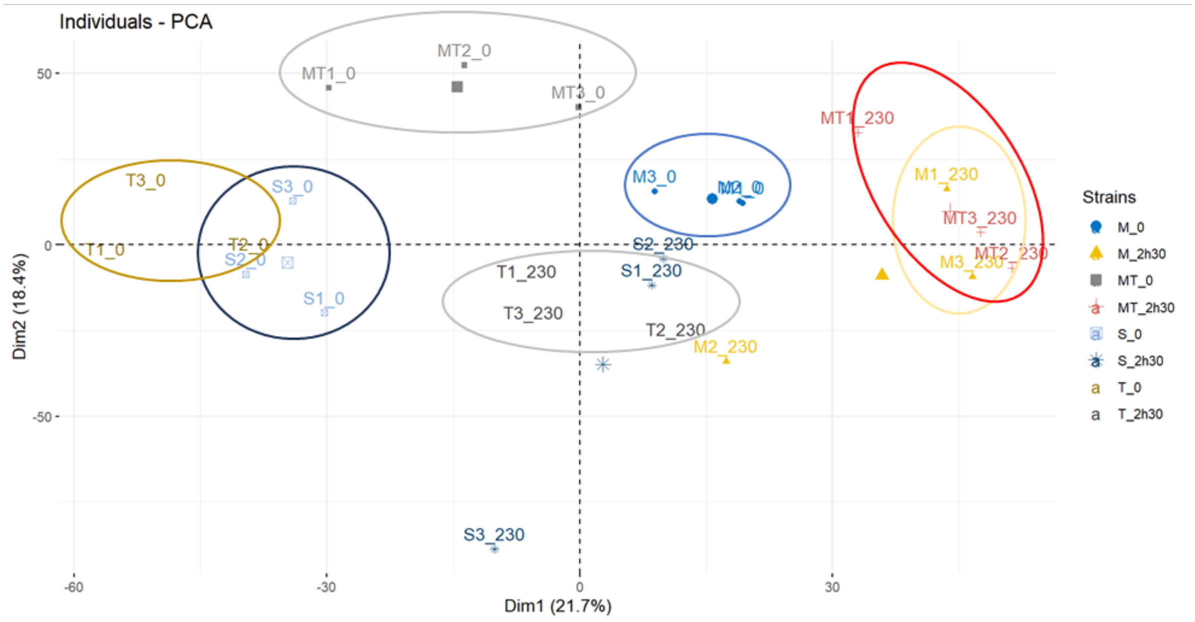

Supplementary Fig. 7: PCA of the protein abundance data of SS, MM, TT, and MMTT strains during the 0 h and 2 h 30 min into sporulation. The analysis identified one of the S288c strain replicates at 2 h 30 min as a potential outlier, which was excluded from further analysis. The source data are provided as a Source data file.

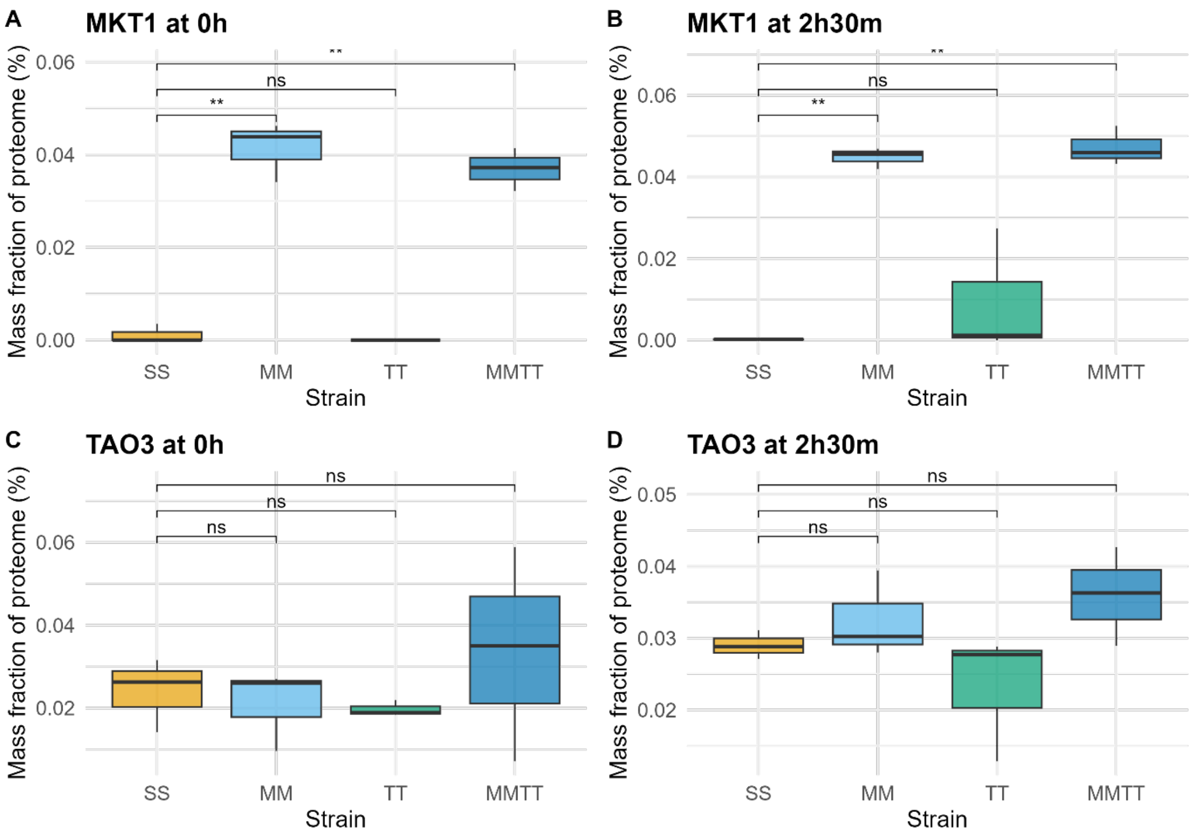

Supplementary Fig. 8: Protein expression levels of *MKT1* and *TAO3* across yeast strains and time points. Boxplots showing the mass fraction of the proteome (%) allocated to the *MKT1* (panels A, B) and *TAO3* (panels C, D) and in four yeast strains (SS, MM, TT, and MMTT) at two time points: 0 h (A, C) and 2 h 30 min (B, D) after sporulation induction. Statistical comparisons were performed using a two-sided unpaired t-test between SS and each of the other strains (MM, TT, MMTT). Significance levels are indicated as follows: ns, not significant; *, $p < 0.05$; **, $p < 0.01$. Source data are provided as a Source Data file.

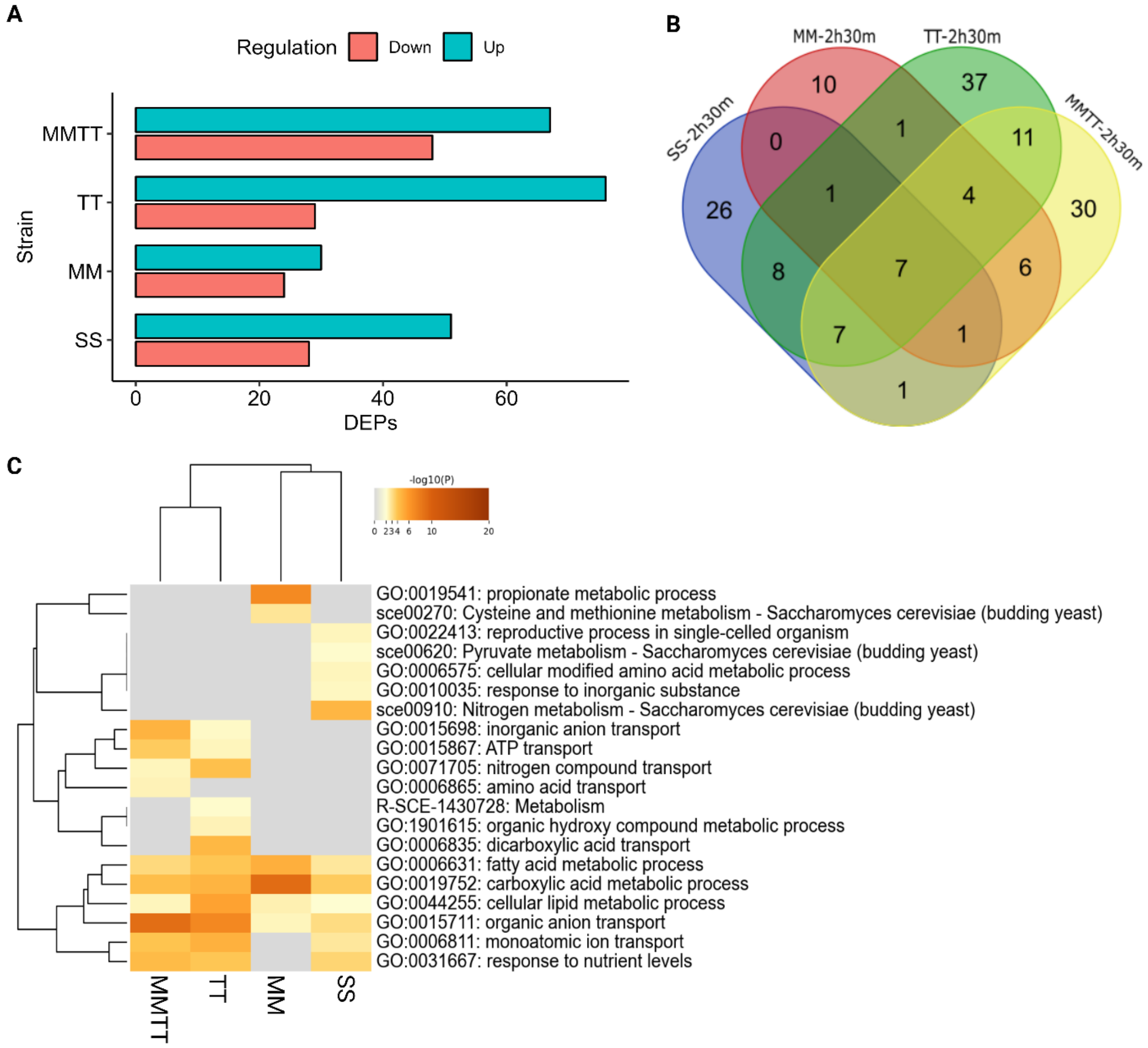

Supplementary Fig. 9: Differentially expressed proteins in the SS, MM, TT, and MMTT strains during the early sporulation phase (2 h 30 min). (A) Bar plots showing the number of significantly differentially expressed genes compared to the initial time point. (B) Venn diagrams depicting the number of proteins specifically upregulated and downregulated identified through comparison of the differentially expressed proteins in the three phases of yeast growth. (C) Enrichment GO-terms for upregulated proteins in SS, MM, TT, and MMTT strains during 2 h 30 min in comparison with 0 h. Enrichment p-values were calculated using a one-sided cumulative hypergeometric test and corrected for multiple comparisons using the Benjamini–Hochberg method (Metascape). Source data are provided as a Source Data file and in Supplementary Data 6.

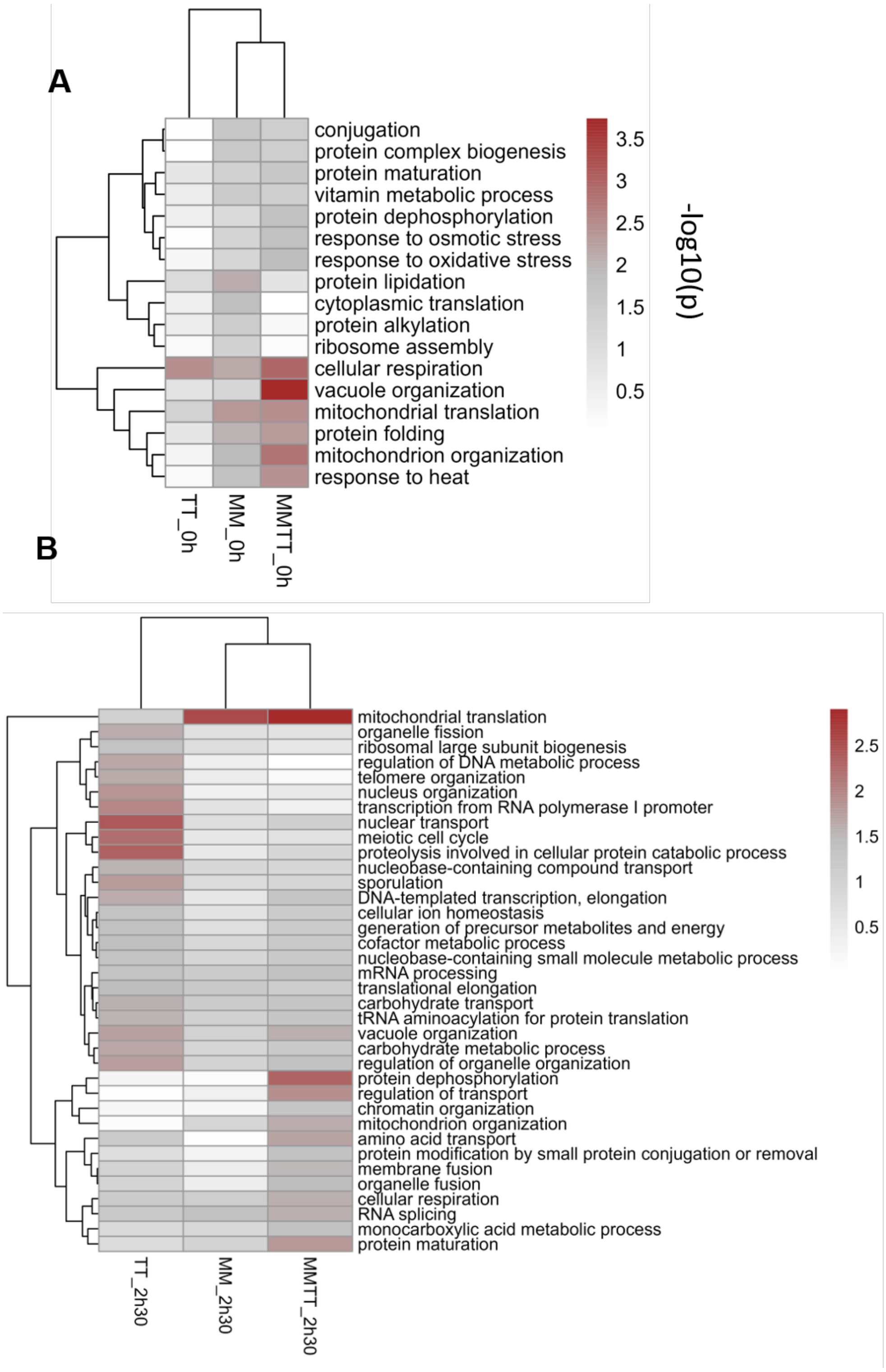

Supplementary Fig. 10: Heatmaps showing the $-\log_{10}(p)$ values from pairwise comparisons of protein allocation between the SS strain and allele replacement strains (MM, TT, and MMTT) for selected GO-mapper terms at (A) 0 h and (B) 2 h 30 min. GO terms with significant differences ($p < 0.05$ in at least one comparison) are displayed. Statistical significance was determined using a two-sided unpaired Student's t-test. Source data are provided as a Source Data file.

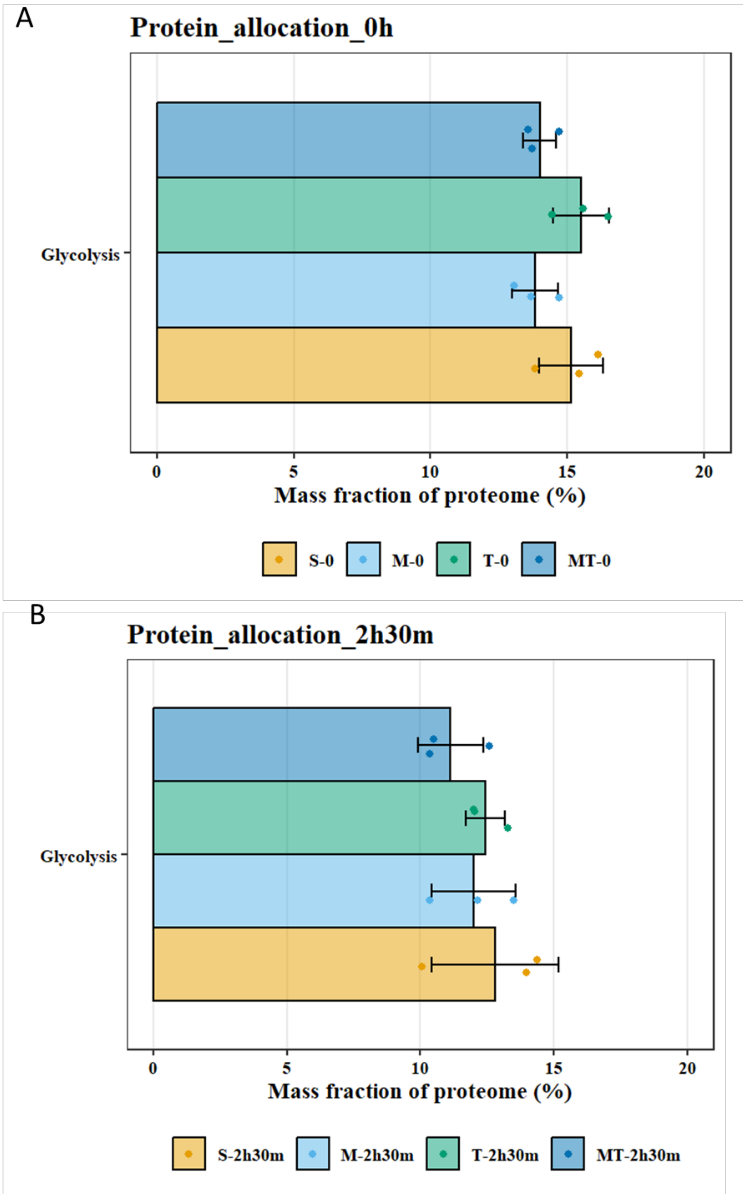

Supplementary Fig. 11: Protein allocation to glycolysis decreases during sporulation in the MMTT strain. Allocation of the whole cellular proteome to the glycolysis pathway in SS, MM, TT, and MMTT during the (A) 0 h and (B) early stage of sporulation (2 h 30 min), calculated as the mean percentage allocation. Data are mean ± SD of three biological replicates. Individual points are represented as dots. Source data are provided as a Source Data file.

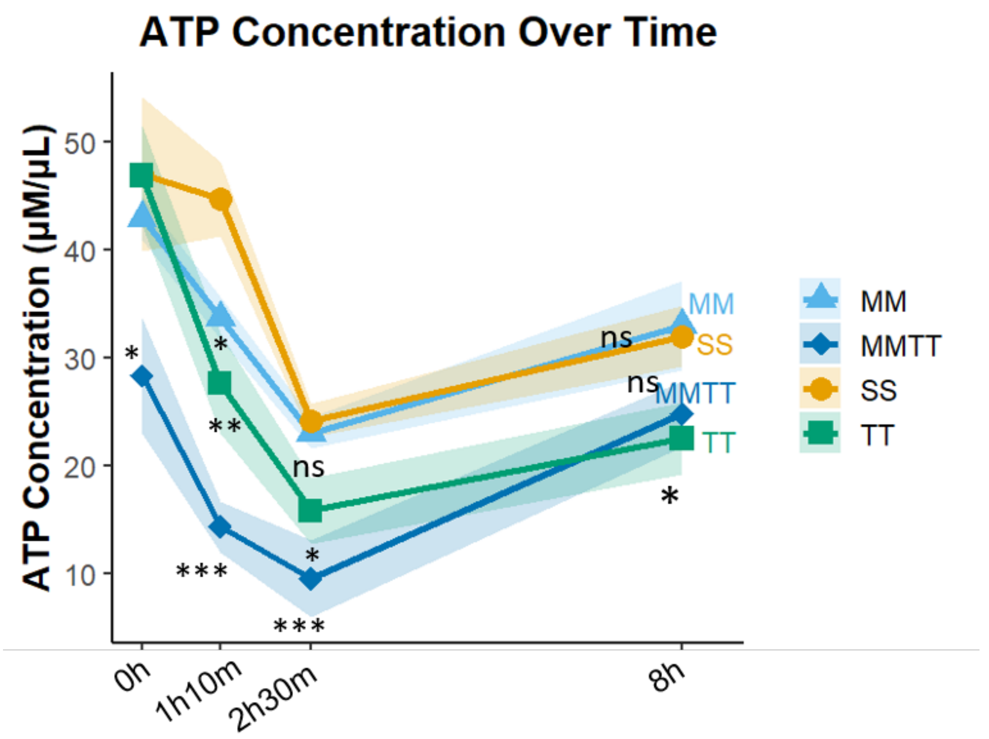

Supplementary Fig. 12: Intracellular ATP concentrations over time in sporulation medium. ATP levels were quantified in strains SS, MM, TT, and MMTT at 0 h, 1.16 h, 2.5 h, and 8 h. Data are presented as mean ± SD from 2 to 3 biological replicates. Statistical significance was assessed using a two-sided unpaired t-test for MM, TT, and MMTT in comparison with the S strain (*p < 0.05, **p < 0.01, ***p < 0.001; ns, not significant) (n = 2 or 3). Source data are provided as a Source Data file.

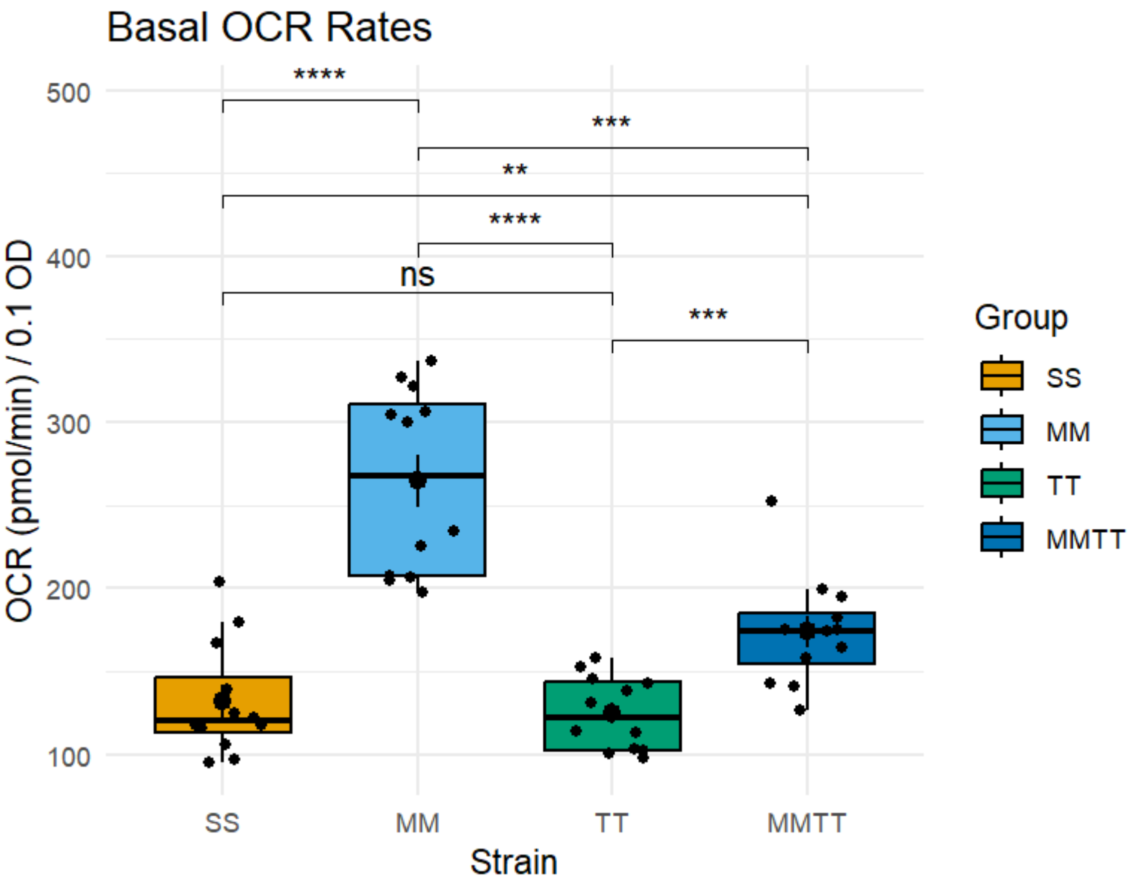

Supplementary Fig. 13: Basal oxygen consumption rate (OCR) across yeast strains. Boxplots show the normalised basal OCR (pmol min^{-1} per 0.1 OD) for each strain: SS, MM, TT, and MMTT. Each point represents an individual three Seahorse measurement under acetate medium conditions. Statistical comparisons were performed using a two-sided pairwise t-test with Bonferroni correction. Significant differences between groups are marked with asterisks (* $p < 0.05$, ** $p < 0.01$, *** $p < 0.001$, **** $p < 0.0001$). Source data are provided as a Source Data file.

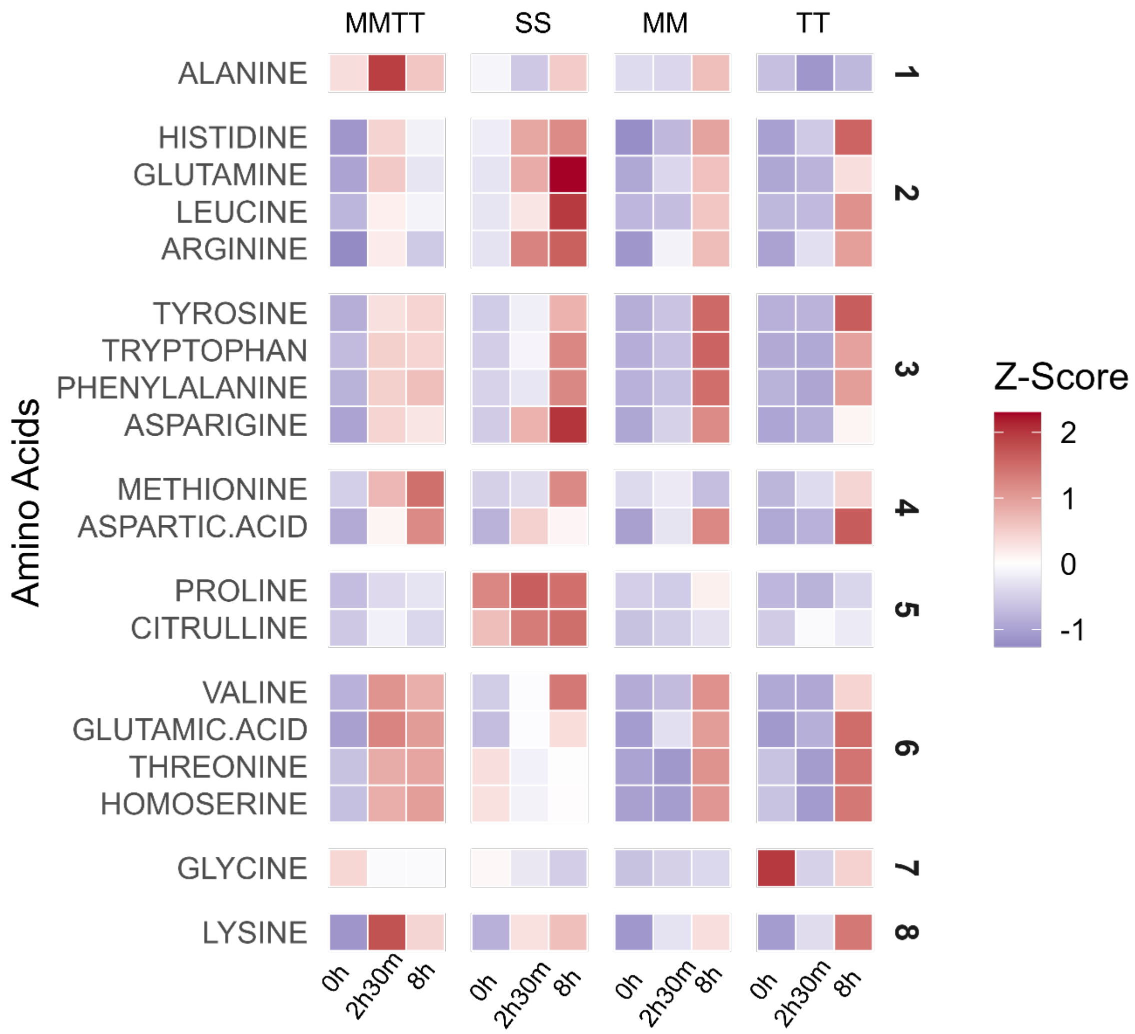

Supplementary Fig. 14: Heatmap showing the z-scores of amino acid intensity values across time points and strains. Amino acids are ordered based on the hierarchical clustering of values in the MMTT strain, keeping time constant. Source data are provided as a Source Data file.

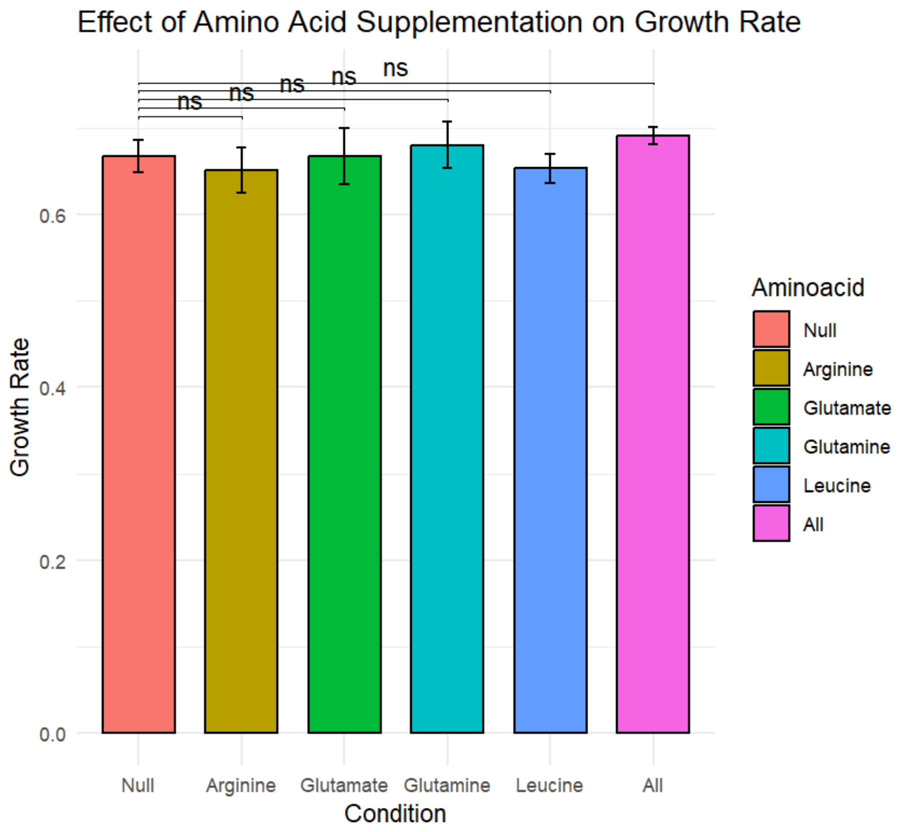

Supplementary Fig. 15: Amino acid supplementation does not rescue the growth defect of MMTT-*arg4*Δ. Bar plot showing the growth rates of the MMTT-*arg4*Δ strain in YPD and YPD supplemented with individual amino acids. Growth rates were measured under each condition, and the mean ± SD is shown. Statistical significance was assessed using a two-sided unpaired t-test comparing each supplemented condition to the YPD control. Significance levels are indicated as follows: ns, not significant; *, $p < 0.05$; **, $p < 0.01$. Source data are provided as a Source Data file.

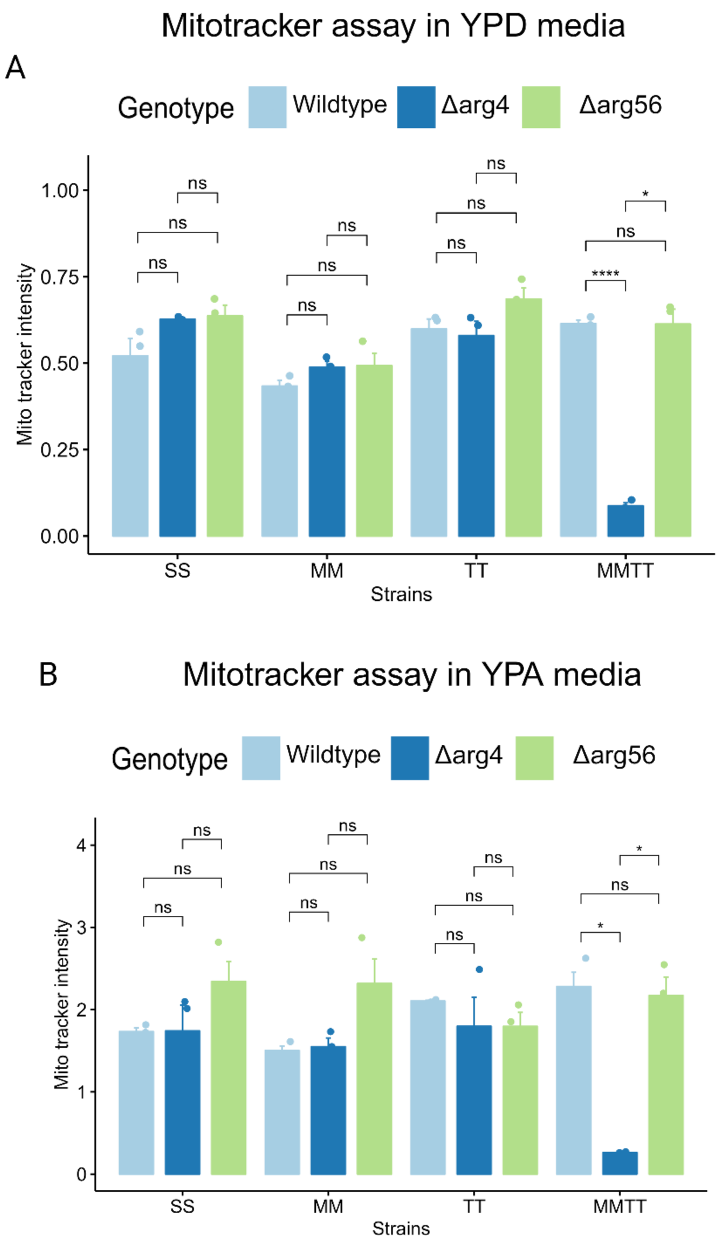

Supplementary Fig. 16: Mitotracker fluorescent assay on haploid S, M, T, and MT and their respective *arg4* and *arg56* deletions after 2 h incubation in (A) YPD medium and (B) YPA medium. P-values were calculated using a two-sided unpaired t-test in the rstatix R package (v.0.7.2). Significance levels are indicated as follows: * $p < 0.05$, ** $p < 0.01$, *** $p < 0.001$, **** $p < 0.0001$, and ns (not significant) for $p > 0.05$. The mean ± S.D. of at least three biological replicates is shown. Source data are provided as a Source Data file.

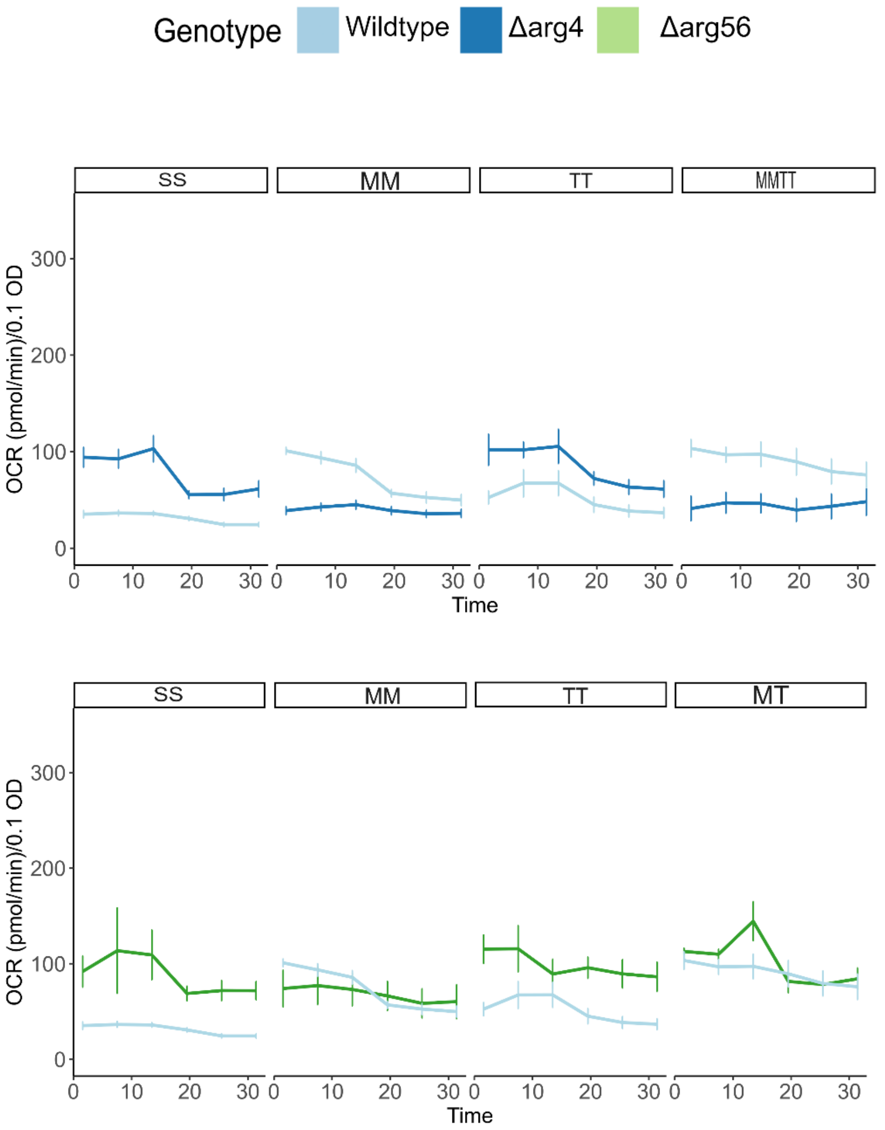

Supplementary Fig. 17: The oxygen consumption rate (OCR) of SS, MM, TT, and MMTT for wild type (n = 4), *arg4Δ* (n = 4), and *arg56Δ* (n = 3) cells was measured using the Seahorse Extracellular Flux 96 Analyzer, grown in glucose media. After three basal measurements, sodium azide was injected into the wells to shut off mitochondrial oxygen consumption, and an additional three sets of measurements were taken. The error bars represent the Mean ± SD. Source data are provided as a Source Data file.

A

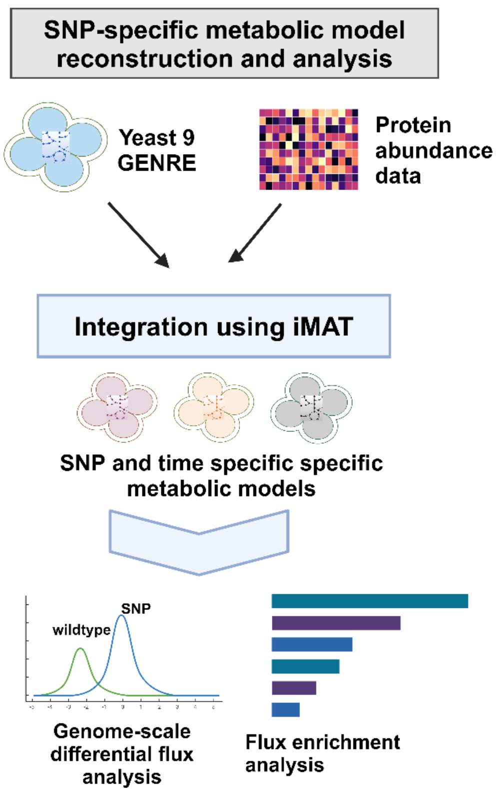

B

Models	Reactions	Genes	Metabolites
SS-0	1222	577	966
MM-0	1232	561	972
TT-0	1191	567	932
MMTT-0	1216	561	959
SS-2h30m	1223	586	962
MM-2h30m	905	374	660
TT-2h30m	922	376	675
MMTT-2h30m	990	406	744

C

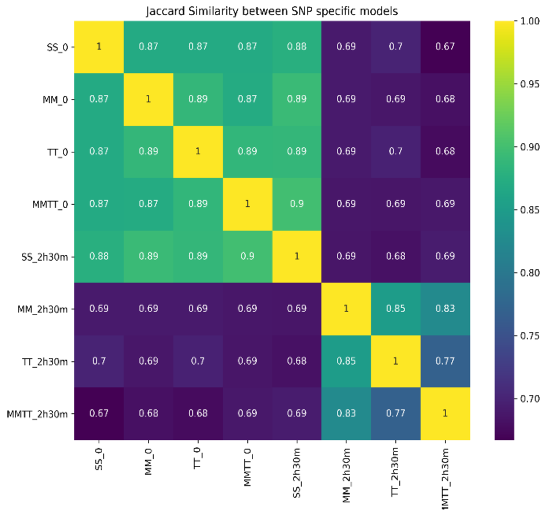

Supplementary Fig. 18: Context-specific models show metabolic heterogeneity during sporulation. (A) Schematic representation of steps involved in generating and analyzing SNP and time-specific models. (B) The number of reactions, genes, and metabolites present in each context-specific model. (C) Jaccard similarity index of context-specific models based on the presence or absence of reactions. Created in BioRender. Sinha, H. (2025) https://BioRender.com/07eizi3

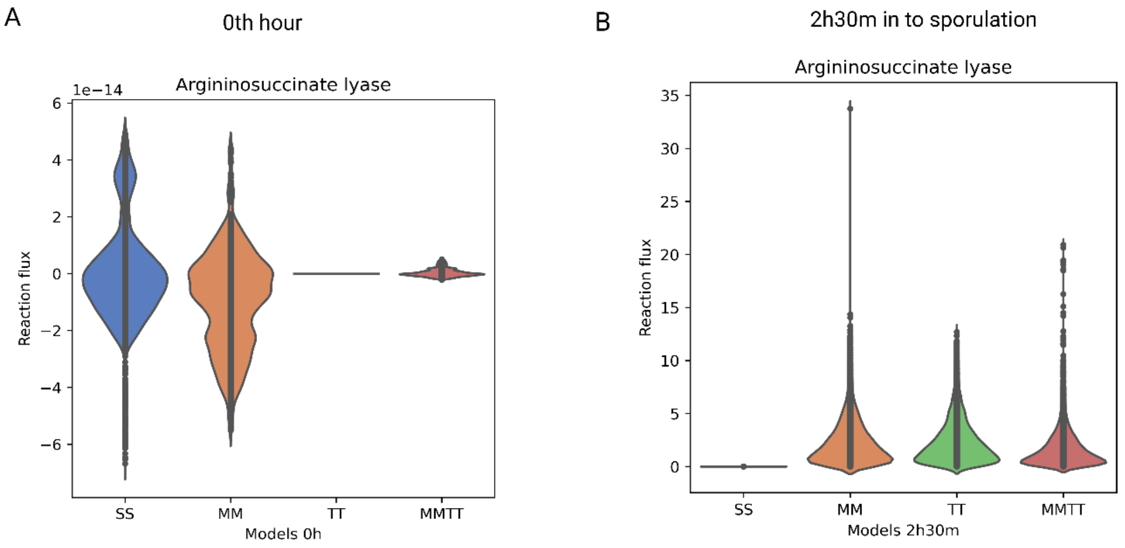

Supplementary Fig. 19. The violin plots represent the flux distribution obtained by optGpSampler, with seed set to 5, for Argininosuccinate lyase (*ARG4*) reaction during the (A) 0 h and (B) 2 h 30 min into sporulation in SS, MM, TT, and MMTT models.

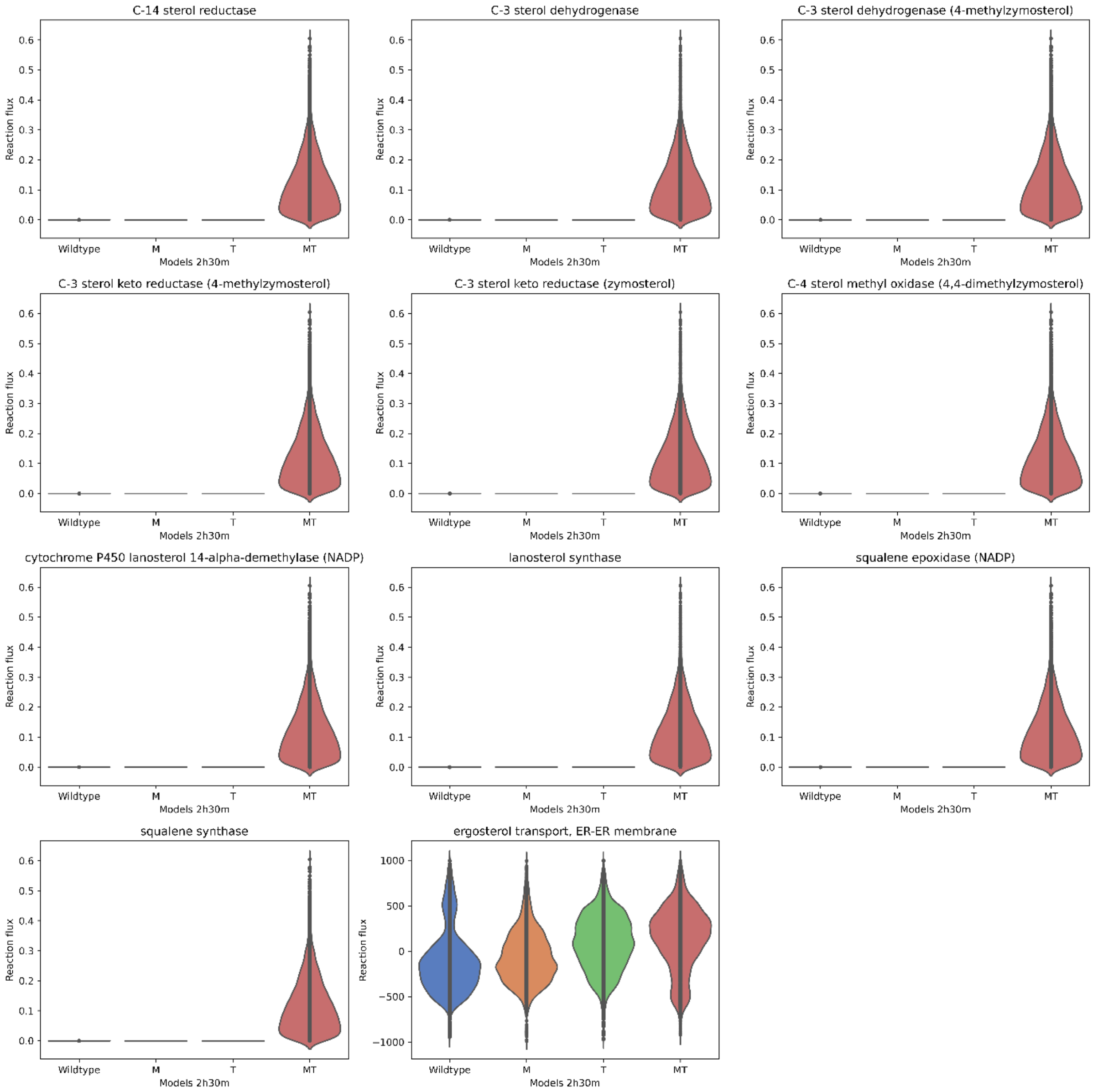

Supplementary Fig. 20. Steroid biosynthesis pathway is differentially activated when *MKT1*^{89G} and *TAO3*^{4477C} interact. The violin plots represent the flux distribution obtained by optGpSampler, with a seed set to 5, for key reactions in the steroid biosynthesis pathway during 2 h 30 min into sporulation in SS, MM, TT, and MMTT models.

SUPPLEMENTARY REFERENCES

1. Xuan, Y. et al. Standardization and harmonization of distributed multi-center proteotype analysis supporting precision medicine studies. *Nat. Commun.* **11**, 5248 (2020).
2. Zhang, C. et al. Yeast9: a consensus genome-scale metabolic model for S. cerevisiae curated by the community. *Mol. Syst. Biol.* **1–17** (2024)
3. Zur, H., Ruppin, E. & Shlomi, T. iMAT: an integrative metabolic analysis tool. *Bioinformatics* **26**, 3140–3142 (2010).
4. Sasikumar, S., Kumar, S. P., Bhatt, N. P. & Sinha, H. Genome-scale metabolic modelling identifies reactions mediated by SNP-SNP interactions associated with yeast sporulation. *npj Syst. Biol. Appl.* **11**, 1–15 (2025).
5. Megchelenbrink, W., Huynen, M. & Marchiori, E. optGpSampler: An Improved Tool for Uniformly Sampling the Solution-Space of Genome-Scale Metabolic Networks. *PLoS One* **9**, e86587 (2014).
6. Ebrahim, A., Lerman, J. A., Palsson, B. O. & Hyduke, D. R. COBRApy: COnstraints-Based Reconstruction and Analysis for Python. *BMC Syst. Biol.* **7**, 74 (2013).
7. Nanda, P. & Ghosh, A. Genome Scale-Differential Flux Analysis reveals deregulation of lung cell metabolism on SARS-CoV-2 infection. *PLoS Comput. Biol.* **17**, e1008860 (2021).
8. Deutschbauer, A. M. & Davis, R. W. Quantitative trait loci mapped to single-nucleotide resolution in yeast. *Nat. Genet.* **37**, 1333–1340 (2005).
9. Gupta, S. et al. Temporal Expression Profiling Identifies Pathways Mediating Effect of Causal Variant on Phenotype. *PLoS Genet.* **11**, 1–23 (2015).
10. Gupta, S. et al. Meiotic interactors of a mitotic gene Tao3 revealed by functional analysis of its rare variant. *G3 (Bethesda)* **6**, 2255–2263 (2016).
