# Supplementary material for: Interaction of genetic variants activates latent metabolic pathways in yeast
Source: Nat Commun. 2025 Aug 27;16:8014. doi: 10.1038/s41467-025-63306-4 (PMC12391433; doi:10.1038/s41467-025-63306-4)
Supplement: Supplementary file 2 — Description of additional supplementary files [file 41467_2025_63306_MOESM2_ESM.pdf]

Description of Additional Supplementary Files

Supplementary Data 1
Differentially expressed genes identified using DESeq2.

Supplementary Data 2
Differentially expressed genes identified using the DESeq2 LRT method.

Supplementary Data 3
List of genes present in each cluster for SS and MMTT strain data identified using DPGP clustering.

Supplementary Data 4

Differentially expressed proteins MM, TT and MMTT strain at 0 h identified in comparison to the SS strain.

Supplementary Data 5

Differentially expressed proteins in MM, TT and MMTT strains at 2 h 30 min identified in comparison to the SS strain

Supplementary Data 6

Differentially expressed proteins in SS, MM, TT and MMTT strains at 2 h 30 min identified compared to the 0th hour of each strain, respectively.

Supplementary Data 7

Raw intensity data of intracellular amino acids obtained for SS, MM, TT and MMTT strains at three timepoints (0 h, 2 h 30 min, 8 h) during sporulation.

Supplementary Data 8

List of upregulated reactions identified using GS-DFA analysis
