# Supplementary material for: Interaction of genetic variants activates latent metabolic pathways in yeast
Source: Nat Commun. 2025 Aug 27;16:8014. doi: 10.1038/s41467-025-63306-4 (PMC12391433; doi:10.1038/s41467-025-63306-4)
Supplement: Supplementary file 11 — Reporting Summary [file 41467_2025_63306_MOESM11_ESM.pdf]

nature portfolio

Corresponding author(s): Himanshu Sinha

Last updated by author(s): Jul 23, 2025

Reporting Summary

Nature Portfolio wishes to improve the reproducibility of the work that we publish. This form provides structure for consistency and transparency in reporting. For further information on Nature Portfolio policies, see our Editorial Policies and the Editorial Policy Checklist.

Statistics

For all statistical analyses, confirm that the following items are present in the figure legend, table legend, main text, or Methods section.

n/a	Confirmed	
☐	☒	The exact sample size (*n*) for each experimental group/condition, given as a discrete number and unit of measurement
☐	☒	A statement on whether measurements were taken from distinct samples or whether the same sample was measured repeatedly
☐	☒	The statistical test(s) used AND whether they are one- or two-sided<br>*Only common tests should be described solely by name; describe more complex techniques in the Methods section.*
☐	☒	A description of all covariates tested
☐	☒	A description of any assumptions or corrections, such as tests of normality and adjustment for multiple comparisons
☐	☒	A full description of the statistical parameters including central tendency (e.g. means) or other basic estimates (e.g. regression coefficient) AND variation (e.g. standard deviation) or associated estimates of uncertainty (e.g. confidence intervals)
☐	☒	For null hypothesis testing, the test statistic (e.g. *F*, *t*, *r*) with confidence intervals, effect sizes, degrees of freedom and *P* value noted<br>*Give P values as exact values whenever suitable.*
☒	☐	For Bayesian analysis, information on the choice of priors and Markov chain Monte Carlo settings
☒	☐	For hierarchical and complex designs, identification of the appropriate level for tests and full reporting of outcomes
☐	☒	Estimates of effect sizes (e.g. Cohen's *d*, Pearson's *r*), indicating how they were calculated

Our web collection on statistics for biologists contains articles on many of the points above.

Software and code

Policy information about availability of computer code

Data collection	Spectronaut v18; xTop algorithm v2.1; Agilent MassHunter Workstation Quantitative Analysis software (version 10.1)
Data analysis	Raw Fastq files were given as input to a customised Snake make pipeline, which performs quality checks using FASTQC and MutiQC and performs Yeast S288c reference genome alignment using STAR aligner. The feature count file was then input into the IDEP 2.0 software. The differential gene expression analysis was performed using the DEseq2 (R package). The temporal gene expression clustering was performed using the DPGP clustering algorithm [https://github.com/PrincetonUniversity/DP_GP_cluster]. The differential protein abundance analysis was performed using a custom R code. The R codes used for analyses are available on Zenodo [https://doi.org/10.5281/zenodo.13917859]. The context-specific metabolic models were generated using the iMAT algorithm available in MatlabR2021b [https://opencobra.github.io/cobratoolbox/stable/index.html]. optgp sampler available in Cobrapy [https://opencobra.github.io/cobrapy/]. rstatix 0.7.2 (R package) for statistical analysis.

For manuscripts utilizing custom algorithms or software that are central to the research but not yet described in published literature, software must be made available to editors and reviewers. We strongly encourage code deposition in a community repository (e.g. GitHub). See the Nature Portfolio guidelines for submitting code & software for further information.

Data

Policy information about availability of data

All manuscripts must include a data availability statement. This statement should provide the following information, where applicable:

- Accession codes, unique identifiers, or web links for publicly available datasets
- A description of any restrictions on data availability
- For clinical datasets or third party data, please ensure that the statement adheres to our policy

Fastq files and raw gene-count data tables are available on Gene Expression Omnibus (GEO) under the accession number GSE278261. Mass spectrometry proteomics data have been deposited to the ProteomeXchange Consortium via the PRIDE partner repository with the dataset identifier PXD056947. The R codes used for analyses are available on Zenodo [https://doi.org/10.5281/zenodo.13917859].

Research involving human participants, their data, or biological material

Policy information about studies with human participants or human data. See also policy information about sex, gender (identity/presentation), and sexual orientation and race, ethnicity and racism.

Reporting on sex and gender	The study did not involve humans.
Reporting on race, ethnicity, or other socially relevant groupings	The study did not involve humans.
Population characteristics	The study did not involve humans.
Recruitment	The study did not involve humans.
Ethics oversight	The study did not involve humans.

Note that full information on the approval of the study protocol must also be provided in the manuscript.

Field-specific reporting

Please select the one below that is the best fit for your research. If you are not sure, read the appropriate sections before making your selection.

☒ Life sciences ☐ Behavioural & social sciences ☐ Ecological, evolutionary & environmental sciences

For a reference copy of the document with all sections, see nature.com/documents/nr-reporting-summary-flat.pdf

Life sciences study design

All studies must disclose on these points even when the disclosure is negative.

Sample size	No sample size calculation was performed. Biological replicates were used in this study because for multi-omics studies it is quite common to perform them in replicates and this is generally considered to be sufficient. All available strains were included in the study.
Data exclusions	We removed outlier samples in RNASeq and Proteomics data.
Replication	Biological replicates were used.
Randomization	Randomization was not necessary in study design.
Blinding	Blinding was not applicable in study design.

Reporting for specific materials, systems and methods

We require information from authors about some types of materials, experimental systems and methods used in many studies. Here, indicate whether each material, system or method listed is relevant to your study. If you are not sure if a list item applies to your research, read the appropriate section before selecting a response.

Materials & experimental systems

n/a	Involved in the study
☒	☐ Antibodies
☒	☐ Eukaryotic cell lines
☒	☐ Palaeontology and archaeology
☒	☐ Animals and other organisms
☒	☐ Clinical data
☒	☐ Dual use research of concern
☒	☐ Plants

Methods

n/a	Involved in the study
☒	☐ ChIP-seq
☒	☐ Flow cytometry
☒	☐ MRI-based neuroimaging

Plants

Seed stocks	The study did not involve plants.
Novel plant genotypes	The study did not involve plants.
Authentication	The study did not involve plants.
